# Microfluidic Fabrication of Monodisperse Microcapsules for Thermo-Triggered Release of Liposoluble Drugs

**DOI:** 10.3390/polym12102200

**Published:** 2020-09-25

**Authors:** Yuanyuan Wang, Yongyue Li, Jinghua Gong, Jinghong Ma

**Affiliations:** State Key Laboratory for Modification of Chemical Fibers and Polymer Materials, College of Materials Science and Engineering, Donghua University, Shanghai 201620, China; yuanyuanhpu@163.com (Y.W.); yueyuexiaochou@163.com (Y.L.); gjh@dhu.edu.cn (J.G.)

**Keywords:** microcapsule, poly(N-isopropylacrylamide-*co*-methacrylic acid), double coaxial microfluidic device, thermo-triggered release, liposoluble drug

## Abstract

Here, we report a novel thermo-triggered-releasing microcapsule for liposoluble drug delivery. Monodisperse microcapsules with a poly(N-isopropylacrylamide-*co*-methacrylic acid) hydrogel shell and an oil core were successfully fabricated by a double coaxial microfluidic device. Fluorescent dye Lumogen Red F300 as a model liposoluble drug was dissolved in the oil core with controllable loading capacity. The volume phase transition temperature (VPTT) of the microcapsule was adjusted by copolymerizing with the hydrophilic methacrylic acid. The in vitro release study demonstrates that the shells shrink, leading to the thermo-triggered release of the model drug from the microcapsules at the environmental temperature above the VPTT, while the swollen hydrogel shells can protect the encapsulated drug from leakage and contamination below the VPTT. The proposed microcapsule is a promising liposoluble drug delivery system with controllable loading and smart thermo-triggered release.

## 1. Introduction

Microcapsules have been widely used in the pharmaceutical industry because they can mask unpleasant drug tastes and odors, control drug release, reduce adverse drug reactions, and prevent undesirable degradation. Researchers have long been exploring tools to deliver drugs to the target site in a controllable way. Considerable efforts have been made to design and fabricate stimuli-sensitive microcapsules or hydrogel microspheres because of their potential applications in the protection of active species [1,2,3], drug delivery [4,5,6,7], diagnostic imaging [8,9], and confined microreactions [10,11]. Stimuli-sensitive microcapsules can automatically detect changes in the temperature and chemical environment around the nidus, and thus controllably release drugs.

Poly(N-isopropylacrylamide) (PNIPA) is a well-known thermo-sensitive polymer with a dramatic phase transition property when the environmental temperature changes across its lower critical solution temperature. PNIPA microcapsules or hydrogels swell at temperatures lower than its volume phase transition temperature (VPTT, about 33 °C) and shrink at temperatures above it [12,13]. Moreover, the VPTT of the PNIPA hydrogel can be regulated by copolymerizing with a hydrophilic monomer such as methacrylic acid (MAA) [14,15,16], which approaches the physiological temperature. Due to such fantastic and reversible thermo-sensitive properties, PNIPA microcapsules have been extensively studied as temperature-triggered systems for the controlled release of drugs and chemicals [17,18].

Over the last two decades, emulsion polymerization and macromolecular self-assembly are prevalent in the preparation of stimuli-sensitive microcapsules or microspheres. Amphiphilic polymers, which can provide “hydrophobic pockets” for the encapsulation of water-insoluble drugs, and effective strategies to control the cargo release have attracted considerable attention [19]. Several blocks and random amphiphilic copolymers were successfully self-assembled into glucose-responsive nanoparticles or thermo-sensitive micelles for loading insulin or hydrophobic drugs, and in vitro release behaviors were quantitatively investigated [19,20,21]. These studies showed that the cargo loading capacity and the release rates were affected by the copolymer structures and hydrophilic−lipophilic balance.

Microfluidic techniques have been developed in recent years. The fabrication of microcapsules or microspheres based on microfluidics has many advantages including uniform size, controllable and diverse morphology, mild preparation condition, and less chemical contamination [22,23]. Xu et al. [6] fabricated monodisperse drug-loaded microparticles based on biodegradable polymers using a microfluidic flow-focusing device. They also investigated the release kinetics of a model amphiphilic drug (bupivacaine). The results showed that the release of the drug from these monodisperse particles exhibited a significantly lower initial burst that was attributed to the uniform distribution of the drug inside the particles. Liu et al. [24] used a microfluidic approach to prepare monodisperse core–shell microcapsules based on a cross-linked chitosan membrane. The microcapsules presented acid-triggered burst release behaviors that could decompose rapidly and release the encapsulated contents in an acidic medium (pH 1.5–4.7). Liu et al. [25] designed a thermo-triggered squirting microcapsule for delivering nanoparticles. The microcapsule was composed of a cross-linked PNIPA hydrogel shell and water-in-oil (W/O) emulsion core with nanoparticles encapsulated in the water phase. The swollen and hydrophilic PNIPA hydrogel membrane could protect the encapsulated nanoparticles below the VPTT and eject them above the VPTT.

Drug molecules can be embedded in microcapsules by physical interactions or chemical bonds. In this paper, we designed a novel thermo-triggered-releasing microcapsule for tunable drug loading. The fluorescent dye Lumogen Red F300 was used as a model liposoluble drug. We used a double coaxial microfluidic device to fabricate monodisperse microcapsules consisting of thermo-sensitive P(NIPA-*co*-MAA) hydrogel shells and oil cores with the model drug encapsulated inside. The drug loading capacity can be easily adjusted by changing the concentration of drugs in the oil core. The releasing behavior of the model drug depends on the swelling or shrinking of the thermo-sensitive P(NIPA-*co*-MAA) hydrogel shell. The state of P(NIPA-*co*-MAA) microcapsules at different temperatures was studied, as well as the in vitro release process of the oil core.

## 2. Experiments

### 2.1. Materials

N-isopropylacrylamide (NIPA) was purchased from Tokyo Chemical Industry Co., Japan and purified by recrystallization with a toluene-cyclohexane mixture (60/40, *v*/*v*) to remove inhibitor prior to use. Methacrylic acid (MAA) was purchased from Sinopharm Chemical Reagent Co., Shanghai, China and purified by vacuum distillation. N,N’-methylene diacrylamide (BIS) and Pluronic F127 were purchased from Sigma-Aldrich Co., St. Louis, MO, USA. Benzoin dimethyl ether (BDK) and Irgacure 2959 were purchased from Aladdin Chemical Co., Shanghai, China. Polyglycerol polyricinoleate (PGPR 90, food-grade) was purchased from Danisco Co., Ltd. (Kunshan, China) Glycerol was purchased from Shanghai Ling Feng Chemical Reagent Co., Shanghai, China. Fluorescent dye Lumogen Red F300 was purchased from Glorious Chemical Co., Baoding, China. Soybean oil (food grade) was purchased from Yihai Kerry Group, Qinhuangdao, China.

### 2.2. Preparation of P(NIPA-co-MAA) Microcapsules

**Figure 1:**
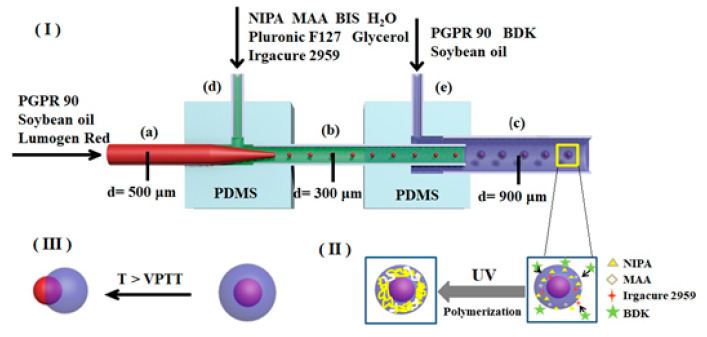
Schematic of P(NIPA-*co*-MAA) microcapsules fabricated by microfluidic device.

According to the literature [26,27], the double coaxial microfluidic device used to fabricate P(NIPA-*co*-MAA) microcapsules was assembled by two PDMS connectors, three capillaries, and two needles (as shown in Figure 1). The production process of the PDMS connection was as follows. A glass capillary with an outer diameter of 1 mm was fixed in the middle of a plastic culture dish. A mixture of PDMS prepolymer and cross-linking agent (mass ratio of 10:1) was poured into the culture dish and then cured for 2 h at 80 °C. After removing the culture dish and the glass capillary, there was a through-hole in the PDMS cube. Then, a hole was drilled vertically above it to obtain a T-channel, so the PDMS connector was made.

A glass capillary with an inner diameter of 500 μm and a 9 gauge needle were inserted into position (a) and (d) of the left PDMS connector, respectively. Then, a glass capillary with an inner diameter of 900 μm and another needle were inserted into position (c) and (e) of the right PDMS connector, respectively. Finally, two PDMS connectors were connected by a glass capillary (b) with an inner diameter of 300 μm. As shown in Figure 1, three glass capillaries used as the inner injection tube (a), the transition tube (b), and the collection tube (c) were coaxially aligned, and two needles ((d) and (e)) were perpendicular to the capillaries for the introduction of fluids.

Three types of solutions were used in this work, including the middle water phase solution, inner oil phase solution, and outer oil phase solution. The middle water phase solution was composed of monomers, a cross-linker, an emulsifier, and a photo-initiator. Monomers NIPA (1.5 g) and MAA (molar percentages in monomers are 0%, 1%, 3%, and 5%, respectively), cross-linker BIS (0.02 g), and emulsifier Pluronic F127 (0.1 g) were dissolved in deionized water (8 mL). Then, glycerol (0.1 g) was added into the solution to adjust the viscosity. Finally, water-soluble photo-initiator Irgacure 2959 (0.01 g/mL, 2 mL) was mixed with this solution. The inner oil phase solution was composed of emulsifier PGPR 90 (3.0 g), Lumogen Red F300 (0.1 g), and soybean oil (100 mL). The outer oil phase solution was composed of oil-soluble photo-initiator BDK (0.2 g), PGPR 90 (5.0 g), and soybean oil (100 mL).

The inner oil phase solution, middle water phase solution, and outer oil phase solution were introduced into the double coaxial microfluidic device separately by syringe pumps (PHD ULTRA, Harvard Apparatus, Holliston, MA, USA), as shown in Figure 1. Their flow rates were 50, 100, and 1900 μL/h, respectively. Based on the coaxial co-flow geometry, O/W emulsions were generated in the transition tube, while O/W/O emulsions were generated in the collection tube. The emulsion droplets out of the microfluidic chip were put in a collection solution with the same composition as the outer oil phase solution. The emulsion droplets were irradiated under a UV LED light source (G365-2, Kunsan Richanghuaxin Co., Kunshan, China) for 30 min to prepare P(NIPA-*co*-MAA) microcapsules. The obtained P(NIPA-*co*-MAA) microcapsules were washed in deionized water many times to remove surface impurities and unreactive monomers, and then stored in deionized water at 20 °C.

To measure the VPTT of the P(NIPA-*co*-MAA) hydrogel, the water phase solution was introduced into the inner injection tube rather than the inner oil phase solution; W/O emulsion droplets were obtained from the microfluidic chip. The polymerization of the W/O emulsion droplets was then carried out similarly to prepare solid P(NIPA-*co*-MAA) microspheres.

The P(NIPA-*co*-MAA) microspheres were analyzed by FTIR (Nicolet 6700, Thermo Fisher, ATR, Waltham, MA, USA).

### 2.3. Morphology and Size of Emulsion Droplets and Microcapsules

The morphology and size of the emulsion droplets and the P(NIPA-*co*-MAA) microcapsules dispersed in the collection solution were characterized by an optical microscope (XSP-17C, Shanghai Optical Instrument Co., Shanghai, China) equipped with a digital CCD camera. The observations were performed at room temperature. The size and size distribution were determined at the same time by randomly selecting 30 samples. The monodispersity of the sample was evaluated based on the coefficient of variation (*CV*) defined as the ratio of the standard deviation of size to its arithmetic mean (*d*) [28].

(1) $$d=\overset{n}{\underset{i=1}{\sum }}d_{i}/n$$

(2) CV=100%×{∑i=1n(di−d)2n−1}12d

where *d_i_* is the diameter of the droplets or microcapsules and *n* is the total number of the samples counted. About 30 samples are counted for obtaining each *CV* value.

The P(NIPA-*co*-MAA) microcapsules were washed with deionized water several times and then dispersed in deionized water at 20 °C to reach the swelling equilibrium. The arithmetic mean (*d*) and coefficient of variation (*CV*) were measured as above.

The microcapsules were quickly frozen in liquid nitrogen and then freeze-dried at −50 °C for 8 h. The surface morphology was observed with a scanning electron microscope (SEM, SU8010, Hitachi Co., Tokyo, Japan).

### 2.4. Thermo-Responsive Phase Transition Behaviors of Solid P(NIPA-co-MAA) Microspheres

Thermo-responsive phase transition behaviors of solid P(NIPA-*co*-MAA) microspheres in deionized water (pH = 6.0) were observed by an optical microscope (BX51, Olympus Co., Tokyo, Japan) equipped with a digital CCD camera and a thermostatic stage system. At least 30 samples were selected to calculate the size of the microspheres, and the thermo-response was characterized by the ratio of the microsphere volume at different temperatures to the swelling equilibrium volume of microspheres at 25 °C (*V*/*V*_0_).

### 2.5. Release Behaviors of P(NIPA-co-MAA) Microcapsules

The release behaviors of the P(NIPA-*co*-MAA) microcapsules were also observed with a BX51 optical microscope. First, the microcapsules were dispersed in deionized water (pH = 6.0) at 25 °C to reach their swelling equilibrium. Then, the solution was heated to different temperatures by the thermostatic stage system of the BX51 optical microscope. Each temperature was held constant for 20 min before taking the photographs.

## 3. Results and Discussion

### 3.1. Fabrication of Monodisperse P(NIPA-co-MAA) Microcapsules

(I) Formation of O/W/O emulsion droplets on the chip, (II) UV-initiated polymerization of collected O/W/O emulsion droplets to form P(NIPA-*co*-MAA) microcapsules, and (III) thermo-triggered release behaviors of P(NIPA-*co*-MAA) microcapsules.

The P(NIPA-*co*-MAA) microcapsules were prepared by a double coaxial microfluidic device. There were two stages in this process, namely, formation of the O/W/O emulsion droplets on the chip and free radical polymerization reaction of the monomers off the chip. Figure 1 shows that the inner oil phase solution and the middle water phase solution were separately pumped into the microfluidic device. When they met in the transition tube, O/W emulsion droplets were formed under the shear force. The interior of the droplets was an oil phase containing the food-grade emulsifier PGPR 90, fluorescent dye Lumogen Red F300 (a model of liposoluble drug), and soybean oil. The droplets were surrounded by an aqueous solution composed of monomer NIPA and MAA, cross-linker BIS, water-soluble photo-initiator Irgacure 2959, emulsifier Pluronic F127, and glycerol. The O/W emulsion droplets subsequently flowed into the collection tube and were emulsified in the outer oil phase solution to form O/W/O emulsion droplets.

The second stage was polymerization of the monomers in the emulsion droplets. The emulsion droplets eluting from the microfluidic chip were placed in a collection solution composed of the oil-soluble photo-initiator BDK, PGPR 90, and soybean oil. The water-soluble photo-initiator Irgacure 2959 initiated the free radical polymerization of monomers NIPA and MAA under ultraviolet light. Simultaneously, the photo-initiator BDK in the collection solution initiated the polymerization of the monomers on the droplet surfaces—this enhanced the reaction speed and controlled the morphology. The monomers in the droplets were gradually polymerized and cross-linked under the combined action of the two initiators. As a result, the uniform core–shell microcapsules with soybean oil as the core and P(NIPA-*co*-MAA) hydrogel as the shell were successfully fabricated. The obtained microcapsules are stable in deionized water, indicating that cross-linking network structures of the P(NIPA-*co*-MAA) shells have been formed via the polymerization reaction.

Lumogen Red F300, a model of liposoluble drugs, was dissolved in the inner core layer. To observe the internal structure of the microcapsule clearly, the dye concentration was set to 100 mg/100 mL. In fact, in the P(NIPA-*co*-MAA) microcapsules prepared by the microfluidic method, the drug loading capacity only depends on its saturated solubility in the core solution. As liposoluble drugs cannot diffuse into the hydrophilic shell, all the dissolved drugs can be encapsulated in microcapsules. This means that loading efficiency is almost 100%. The cargo is only exposed to the edible oil and the food-grade emulsifier in the microfluidic preparation process, which prevents chemical contamination.

**Figure 2:**
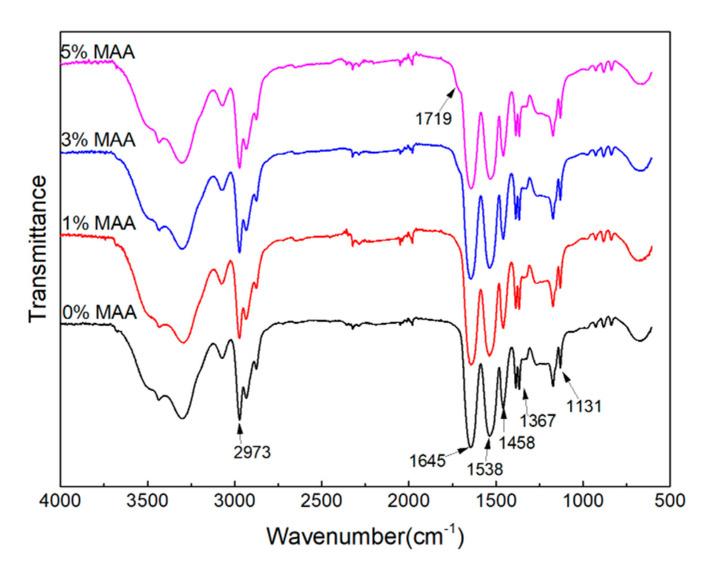
FTIR spectra of P(NIPA-*co*-MAA) microcapsules with different MAA contents.

The P(NIPA-*co*-MAA) microcapsules were characterized by FTIR. As shown in Figure 2, the spectra exhibit main peaks at 1645 and 1538 cm^−1^ corresponding to the characteristic peaks for amide I and amide II, respectively. There is also a characteristic peak at 1719 cm^−1^ for the carbonyl group of MAA, although it is not obvious, due to the low MMA content. The FTIR analysis indicates the successful formation of P(NIPA-*co*-MAA).

### 3.2. Morphology and Size Distribution of O/W/O Emulsion Droplets and P(NIPA-co-MAA) Microcapsules

**Figure 3:**
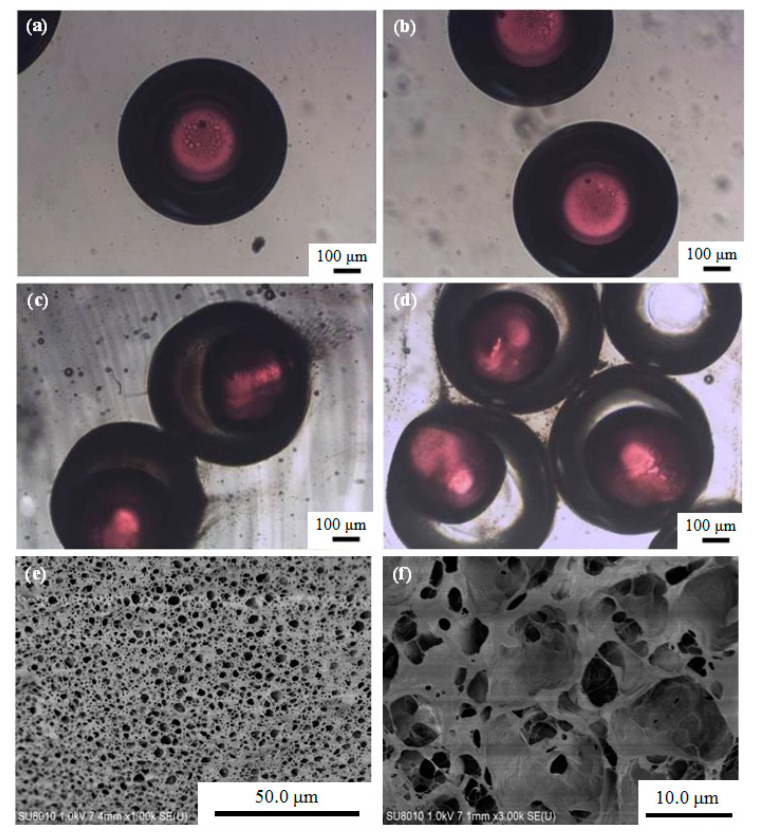
(**a**,**b**) Optical microscopy photographs of O/W/O emulsion droplets, (**c**,**d**) optical microscopy photographs of P(NIPA-*co*-MAA) microcapsules in collection solution, and (**e**) SEM image of the surface morphology of P(NIPA-*co*-MAA) shells. (**f**) SEM image of the internal morphology of P(NIPA-*co*-MAA) shells. The MAA content of the sample is 3 mol.%.

Optical microscopy photographs of the O/W/O emulsion droplets and the P(NIPA-*co*-MAA) microcapsules in the collection solution are shown in Figure 3. Figure 3a,b show the morphology of the O/W/O emulsion droplets. The core–shell structure can be seen based on the fluorescent dyes in the core. The size of the O/W/O emulsion droplets is homogeneous. The mean diameters of the core and shell of O/W/O emulsion droplets are 334.7 and 634.4 μm, respectively. The *CV* values for the core and shell are 1.01% and 2.32%, indicating that monodisperse emulsion droplets were obtained by the microfluidic method.

The morphology of the P(NIPA-*co*-MAA) microcapsules in the collection solution can be found in Figure 3c,d. The mean diameters of the core and shell of the microcapsules are 391.9 and 626.4 μm; their *CV* values are 3.01% and 3.63%, respectively. Versus the O/W/O emulsion droplets, the core size of the microcapsules increased, and the shell size was slightly reduced. The inner oil cores also deformed somewhat. The free radical polymerization of monomers in the shell was exothermic, which probably led to shrinkage of the thermo-sensitive P(NIPA-*co*-MAA) hydrogel shell—this squeezed the inner oil cores.

The surface and internal morphologies of the P(NIPA-*co*-MAA) hydrogel shells were also observed by SEM. The hydrogel shell has an interconnected porous structure (Figure 3e,f). The pore sizes are heterogeneous ranging from hundreds of nanometers to several micrometers, which further confirms the formation of a typical cross-linked hydrogel network.

### 3.3. Thermo-Responsive Phase Transition Behavior of Microcapsules

**Figure 4:**
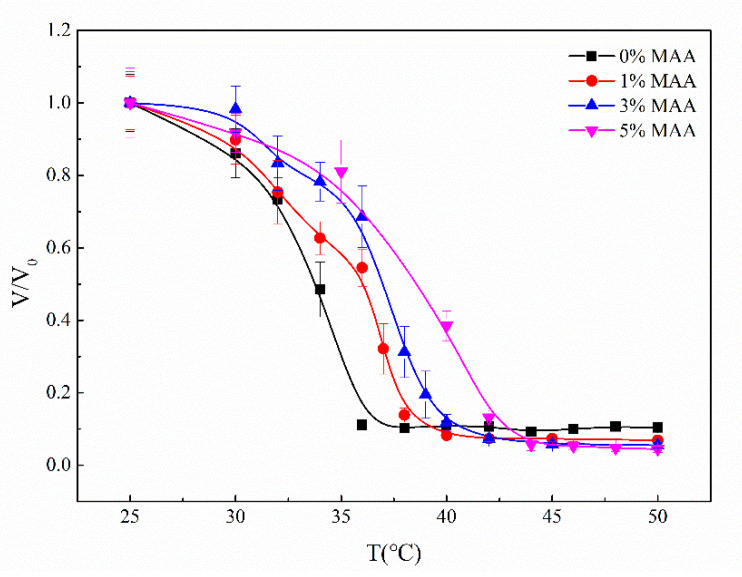
Effect of temperature on the volume ratio of solid P(NIPA-*co*-MAA) hydrogel microspheres with different MAA molar percentages.

The volume transition temperature (VPTT) refers to the temperature at which the hydrogel rapidly shrinks or swells. Solid P(NIPA-*co*-MAA) hydrogel microspheres were prepared to evaluate the VPTT of the microcapsules. Figure 4 shows the effect of temperature on the volume ratio of solid P(NIPA-*co*-MAA) microspheres with different MAA contents. The most obvious change in the volume ratio is the VPTT; the VPTT of the P(NIPA-*co*-MAA) hydrogel microspheres enhances as the molar percentages of MAA increase. For example, the VPTT of PNIPA microspheres is 33 °C, but rises to 37 °C when the MAA content is 3 mol.%. Below the VPTT, the relaxed P(NIPAM-*co*-MAA) chains allow the microcapsules to swell. Once the temperature is above VPTT, P(NIPAM-*co*-MAA) chains pass through the coil-to-globule transition and the network shrinks. The VPTT of microcapsules can be adjusted around the physiological temperature, so it can be used as a temperature-triggered system to control drug release.

**Figure 5:**
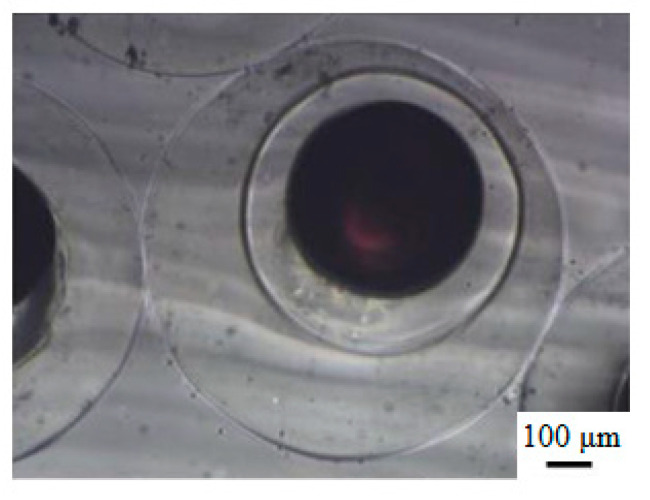
Optical microscopy photographs of P(NIPA-*co*-MAA) microcapsules in deionized water at 20 °C. The MAA content is 3 mol.%.

Figure 5 shows optical microscopy photographs of the P(NIPA-*co*-MAA) microcapsules in deionized water at 20 °C. The mean diameters of the inner core and outer shell are 439.4 and 778.9 μm, and their *CV* values are 1.82% and 2.33%, respectively. The microcapsule size in the deionized water increases distinctly versus the microcapsule in the collection solution. In deionized water at 20 °C (lower than VPTT), the P(NIPA-*co*-MAA) hydrogel shell can absorb water and swell, increasing in microcapsule size. By contrast, the microcapsules do not change their volume in the oil collection solution, because of the hydrophilic and oleophobic nature of the P(NIPA-*co*-MAA) hydrogel shell.

### 3.4. Thermo-Triggered Release Behaviors of P(NIPA-co-MAA) Microcapsules

P(NIPA-*co*-MAA) microcapsules have thermo-sensitive hydrogel shells: Their swelling and shrinking behaviors rely on the temperature. The core is soybean oil loaded with the fluorescent dye Lumogen Red as a model liposoluble drug. The incompatibility of the liposoluble substances with the hydrophilic P(NIPA-*co*-MAA) hydrogel shell makes it impossible for the liposoluble substances to pass through the hydrophilic shell via diffusion or solution even if there is a concentration gradient. This ensures that the liposoluble drugs encapsulated in these microcapsules will not be leaked or contaminated during storage or transportation when the temperature is below the VPTT of the P(NIPA-*co*-MAA) hydrogel shell. However, the shells of the microcapsules will shrink once the environmental temperature rises above the VPTT, and the cargo will release.

**Figure 6:**
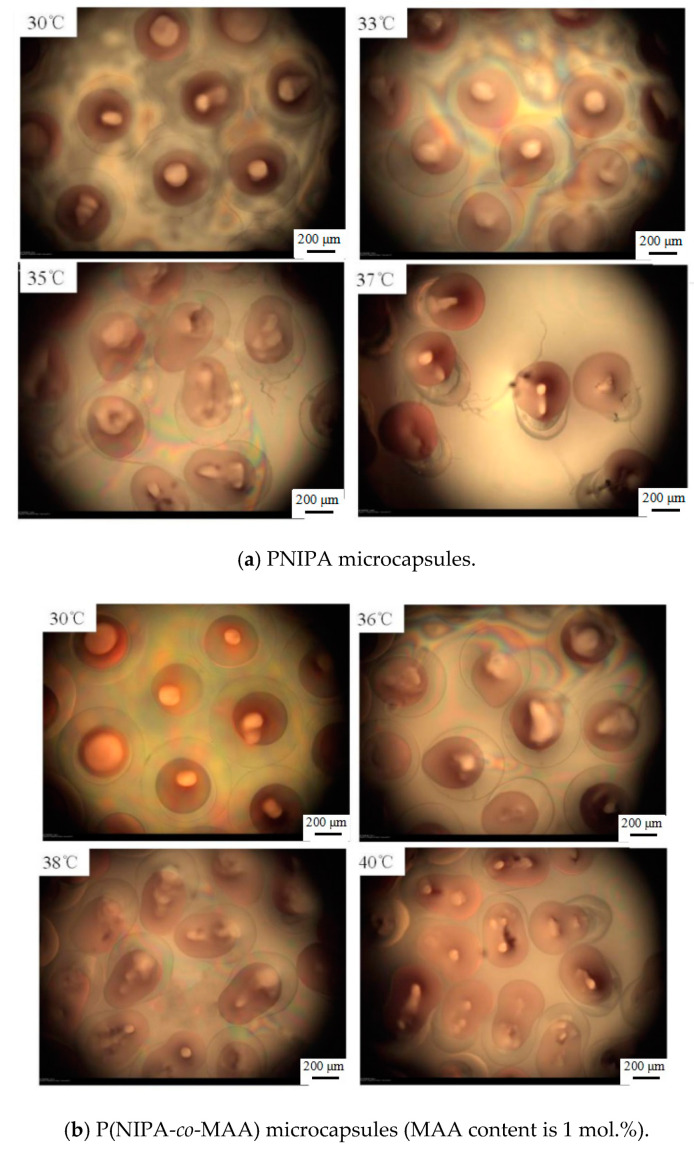

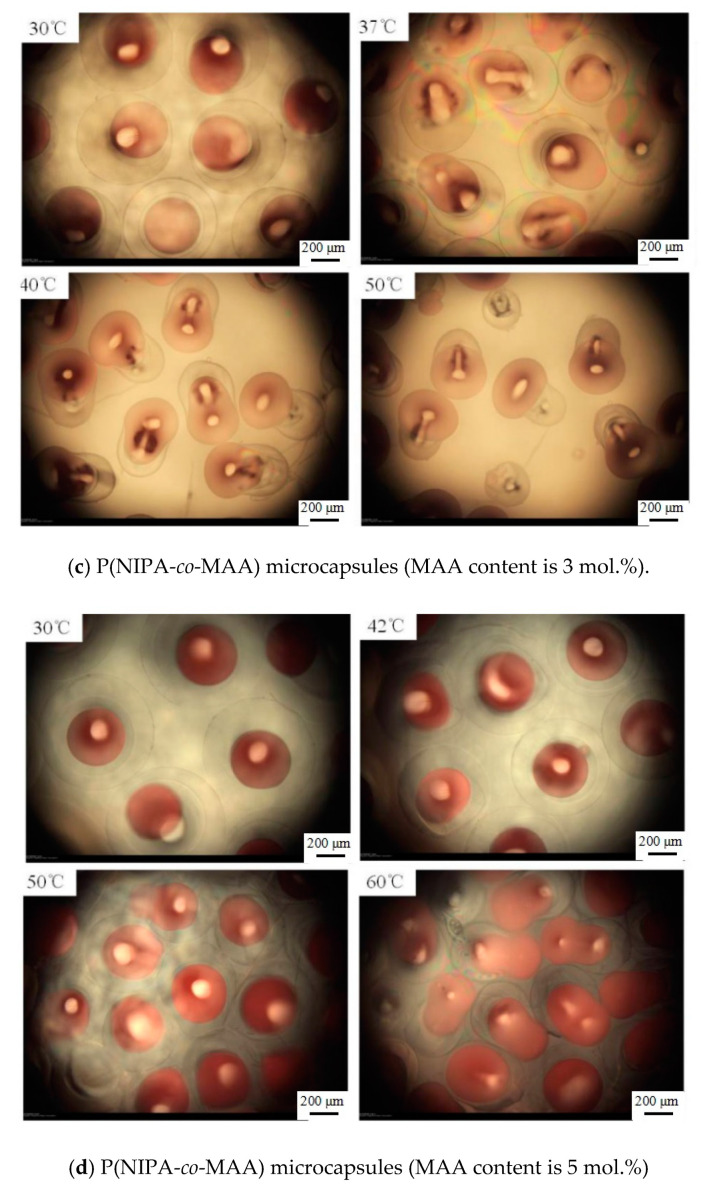
Optical microscopy photographs of release behaviors of P(NIPA-*co*-MAA) microcapsules with different MAA contents in aqueous solution at different temperatures. The scale bar is 200 μm.

Figure 6 shows microscopy photographs of the P(NIPA-*co*-MAA) microcapsules in aqueous solution at different temperatures. The red dye was encapsulated in the inner oil core at temperatures lower than the VPTT of shell hydrogels. Raising the temperature to the VPTT, the microcapsules began to shrink due to their thermo-sensitivity, and the inner oil core was squeezed into an ellipsoidal shape. When further increasing the temperature, the liquid pressure inside the microcapsules kept increasing because of the incompatibility between the oil core and the hydrophilic shell. The hydrogel shell ruptured suddenly when the internal pressure exceeded a critical value; thus, the oil cores were ejected from the microcapsules [25]. As liposoluble drugs were dissolved in the oil solution, it could be inferred that almost 100% drugs released from the microcapsules along with the oil cores.

It is also clearly seen that the release temperature of the oil core is closely related to the VPTT of P(NIPA-*co*-MAA) shells, which depends on the MAA contents. For example, the oil core started to squeeze out of the PNIPA microcapsule at 33 °C, while rising to 37 °C for the P(NIPA-*co*-MAA) shells with 3% MAA. It is conducive for avoiding rapid release of the drug from microcapsules after entering the body. It is also possible to release the drug in a target site, which can be induced by increased temperature.

## 4. Conclusions

Monodisperse microcapsules with a thermo-sensitive P(NIPA-*co*-MAA) hydrogel shell and oil core were successfully fabricated by a double coaxial microfluidic device. The oil core can be used to encapsulate lipophilic drug molecules. The advantage is that the drug loading capacity in the core can be easily regulated to achieve ideal drug entrapment efficiencies. The VPTT values of P(NIPA-*co*-MAA) microspheres can be adjusted by copolymerization with a MAA monomer. The VPTT increases to 37 °C when the MAA content is 3 mol.%. The release behaviors of a model of lipophilic drugs in the inner core depend on the swelling and shrinking of the P(NIPA-*co*-MAA) hydrogel shell. The swollen and hydrophilic hydrogel shell of the microcapsule can protect the encapsulated drug when the environmental temperature is below the VPTT. Increasing the temperature above the VPTT causes the shell to rapidly shrink, which leads to thermo-triggered drug release from the microcapsule.
